# A novel isoform of cryptochrome 4 (Cry4b) is expressed in the retina of a night-migratory songbird

**DOI:** 10.1038/s41598-020-72579-2

**Published:** 2020-09-25

**Authors:** Angelika Einwich, Karin Dedek, Pranav Kumar Seth, Sascha Laubinger, Henrik Mouritsen

**Affiliations:** 1 Institute for Biology and Environmental Sciences, Neurosensorics/Animal Navigation, Carl-von-Ossietzky-Universität Oldenburg, Oldenburg, Germany; 2 Research Centre for Neurosensory Sciences, Carl-von-Ossietzky-Universität Oldenburg, Oldenburg, Germany; 3 Institute for Biology and Environmental Sciences, Evolutionäre Genetik der Pflanzen, Carl-von-Ossietzky-Universität Oldenburg, Oldenburg, Germany

**Keywords:** Biochemistry, Molecular biology

## Abstract

The primary sensory molecule underlying light-dependent magnetic compass orientation in migratory birds has still not been identified. The cryptochromes are the only known class of vertebrate proteins which could mediate this mechanism in the avian retina. Cryptochrome 4 of the night-migratory songbird the European robin (*Erithacus rubecula*; erCry4) has several of the properties needed to be the primary magnetoreceptor in the avian eye. Here, we report on the identification of a novel isoform of erCry4, which we named erCry4b. Cry4b includes an additional exon of 29 amino acids compared to the previously described form of Cry4, now called Cry4a. When comparing the retinal circadian mRNA expression pattern of the already known isoform *erCry4a* and the novel *erCry4b* isoform, we find that *erCry4a* is stably expressed throughout day and night, whereas *erCry4b* shows a diurnal mRNA oscillation. The differential characteristics of the two erCry4 isoforms regarding their 24-h rhythmicity in mRNA expression leads us to suggest that they might have different functions. Based on the 24-h expression pattern, erCry4a remains the more likely cryptochrome to be involved in radical-pair-based magnetoreception, but at the present time, an involvement of erCry4b cannot be excluded.

## Introduction

It is well known that migratory birds use directional information from the Earth’s magnetic field in addition to several other cues to orient and navigate often many thousands of kilometres during their long journeys^1,2^. Already more than 160 years ago, Alexander Theodor von Middendorff suggested that the geomagnetic field plays a role in guiding birds^3^, but only in the 1960s, the use of the magnetic compass was demonstrated experimentally in a night-migratory songbird^4,5^. Since then, a better understanding of the magnetic compass of night-migratory songbirds has been reached: It is an inclination compass^6,7^, located in the retina^8^ of both eyes^9–11^, and dependent on the wavelengths of the surrounding light^12–15^. Magnetic compass information is processed in the thalamofugal visual pathway^16^ at night^17^, and a brain area named “Cluster N” is required for magnetic compass orientation in night-migratory songbirds^8,18,19^. It has also been reported that some non-migratory birds might be able to use a magnetic compass^20–23^ (but see refs.^1,24,25^).

The magnetic compass of night-migratory songbirds is most probably based on a light-dependent quantum mechanism involving radical pairs^26–32^, which is also in line with reports that radio-frequency oscillating magnetic fields disrupt the magnetic compass^27,33–35^. Cryptochromes, a class of photolyase-related flavoproteins first discovered in the plant *A. thaliana*^36^, are considered to be the primary magnetoreceptors in night-migratory songbirds^27,31^, since they are the only proteins known in the avian retina that are able to build radical pairs upon photoexcitation^28,37–40^. Cryptochromes are found in most plants and animals, where they seem to fulfil a wide variety of different functions. In some organisms, they are core elements of the circadian clock^41–43^ and cryptochromes are also reported to be involved in the response to DNA damage, cancer biology and metabolic signalling (for a review, see ref.^44^). Four cryptochrome proteins have been identified to date in the retina of migratory birds: Cryptochrome (Cry) 1a is located in UV/V cones of photoreceptor cells^45^, Cry1b was detected in photoreceptor inner segments, the ganglion cell layer and displaced ganglion cells^46,47^, Cry2 was found associated to the cell nuclei and is most probably involved in the circadian clock^48,49^, and Cry4 was detected in photoreceptor double cones and long-wavelength-sensitive single cones^50^. The *Cry4* gene, first identified in zebrafish^51^ and partially characterised in chicken^52, 53^ and European robin^24^, has, in addition to fish and birds, so far only been found in amphibians^54^ and reptiles^55^, which comprise the four animal classes with a well-documented magnetic sense^1,56–61^. Cry4 is suggested to be the most likely candidate for being the magnetoreceptive protein: Not only is Cry4 of night-migratory European robins (*Erithacus rubecula*) located in a very suitable site for magnetoreception^31^, but it is also upregulated during the migratory season and shows only weak 24-h rhythmicity^50^. Even more importantly, stably bound FAD in vitro has been seen in Cry4 from the chicken^62,63^ and the pigeon^64–66^ and stably bound FAD in vivo is a crucial prerequisite for a protein potentially acting as a magnetoreceptor^27,31^. Furthermore, Cry4 has been suggested to interact with the long-wavelength opsin, iodopsin^67^. Iodopsin is located exclusively in the double cones and long-wavelength single cones, which is where Cry4 is primarily located within the retina of the European robin^50^, and which would seem to be a very suitable site for magnetoreception^31^. Since over 90% of all human protein coding genes produce multiple mRNA isoforms (or splice variants)^68^ and two variants of the *Cry1* gene, *Cry1a* and *Cry1b*, have already been found, we were interested if there might also be more than one isoform of avian *Cry4*. Here, we report that *erCry4* is expressed in at least two splice variants: In addition to the already known *erCry4* isoform, now called *erCry4a*, the novel isoform, called *erCry4b*, possesses one additional exon located within the DNA photolyase homology region. We found the *Cry4b* isoform in the retina from several other bird species and characterised the two isoforms concerning their 24-h mRNA expression and tissue distribution in the European robin.

## Results

### *Cry4b* is a splice variant of avian *Cry4*

**Figure 1:**
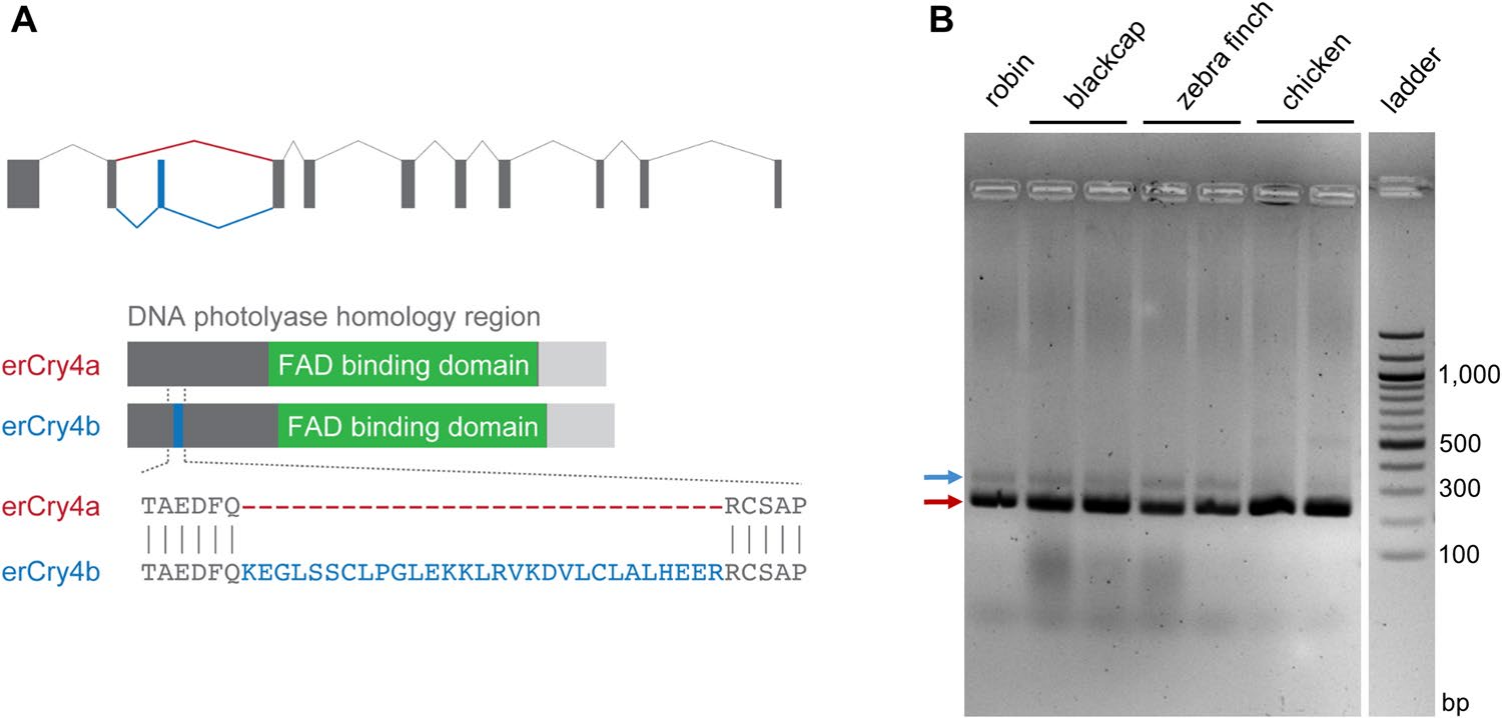
Avian cryptochrome 4 is expressed in two different isoforms. **(A)** Comparison of erCry4a and erCry4b from the European robin. The *erCry4* gene is composed of eleven exons, with the inclusion of exon 3 (blue; nucleotide position 532–618, amino acid position 178–206) being specific for *erCry4b*. The DNA photolyase homology region (spanning amino acids 5–504) and the FAD binding domain therein (amino acids 314–513) are indicated in dark grey and green, respectively. Note that the gene model depicts *Cry4* from the start to the stop codon without the 5′UTR and 3′UTR regions included. **(B)** The isoforms *Cry4a* (red arrow) and *Cry4b* (blue arrow) are expressed in the retinae from different songbird species (European robin, Eurasian blackcap and zebra finch) and possibly also in the domestic chicken. (**A**) was created using Adobe Illustrator, version CS3 (www.adobe.com).

By analysis of the RT-PCR fragment length and subsequent Sanger sequencing of retinal cDNA of the European robin, we detected a novel isoform of *erCry4*, which we named *erCry4b*. In contrast to *erCry4* (now named *erCry4a*), the *erCry4b* mRNA comprises an additional exon of 87 nucleotides (nt) length (29 amino acids, aa) at nt position 532 to 618, which is characteristic for *erCry4b* (Fig. 1A, blue). This newly discovered exon is formed by retention of exon 3 of the *erCry4* gene in the *erCry4b* isoform. The open reading frame (ORF) for the *erCry4b* isoform is composed of eleven exons and has a total length of 1671 nt (556 aa). The mRNA sequence of *erCry4b* is deposited under the GenBank accession number MN709784. By RT-PCR, we also detected the two isoforms expressed in the retinae from the Eurasian blackcap (*Sylvia atricapilla*) and the zebra finch (*Taeniopygia guttata*) (Fig. 1B). In the domestic chicken, we only observed a very faint band of the appropriate size for the *Cry4b* isoform, which suggests that Cry4b might also be expressed in the chicken, but possibly to a much lower extent (Fig. 1B). When subjecting the novel erCry4b-specific exon to a genomic BLAST database search (https://blast.ncbi.nlm.nih.gov/Blast.cgi), we received positive hits for a number of other listed bird species (Table 1). This suggests that the Cry4b isoform is most likely conserved among birds.

**Table 1:** Positive hits of the *erCry4b*-specific exon 3 in BLAST databases.

Species	Bird order	BLAST database (algorithm)	Sequence ID	E value	Query cover (%)	Score
*Anas platyrhynchos*	Anseriformes	wgs (discontiguous megablast)	RHJV01000027.1 NOIJ01000157.1 ADON01119725.1	5e−20	88	102
*Chaetura pelagica*	Apodiformes	wgs (blastn)	AVOS01001646.1	2e−11	100	71.6
*Apteryx australis mantelli*	Apterygiformes	refseq_genomes (discontiguos megablast)	NW_013992250.1	6e−19	100	96.9
*Calidris pugnax*	Charadriiformes	wgs (blastn)	LDEH01010997.1 CZLE01004410.1	4e−23	95	111
*Charadrius vociferus*	Charadriiformes	wgs (blastn)	JMFX02021229.1	5e−25	100	117
*Leptosomus discolor*	Leptosomiformes	wgs (discontiguous megablast)	JJRK01058655.1	1e−26	100	122
*Opisthocomus hoazin*	Opisthocomiformes	wgs (blastn)	JMFL01098842.1	3e−22	100	108
*Acridotheres javanicus*	Passeriformes	wgs (megablast)	PEJO01000078.1	6e−37	100	156
*Camarhynchus parvulus*	Passeriformes	wgs (megablast)	CADCXG010000210.1 CABFNF010000103.1	7e−24	94	113
*Corvus brachyrhynchos*	Passeriformes	wgs (megablast)	JMFN01044619.1	6e−37	100	156
*Corvus cornix cornix*	Passeriformes	wgs (megablast)	MVNZ02000042.1 JPSR03000044.1	1e−36	100	156
*Corvus hawaiiensis*	Passeriformes	wgs, megablast	QORP01000550.1	8e−37	100	156
*Cyanistes caeruleus*	Passeriformes	wgs (megablast)	PDCF01000051.1 OBHQ01000051.1	8e−24	100	113
*Ficedula albicollis*	Passeriformes	wgs (megablast)	AGTO02005649.1	1e−38	100	161
*Geospiza fortis*	Passeriformes	wgs (megablast)	AKZB01054642.1	1e−24	95	115
*Parus major*	Passeriformes	wgs (megablast)	JRXK01014523.1	2e−31	100	137
*Phylloscopus trochiloides trochiloides*	Passeriformes	wgs (megablast)	LXOZ01163281.1	5e−32	100	139
*Phylloscopus trochiloides viridanus*	Passeriformes	wgs (megablast)	LXPA01028412.1	6e−32	100	139
*Pseudopodoces humilis*	Passeriformes	wgs (megablast)	VKJK01000002.1 ANZD01016806.1	1e−29	100	132
*Serinus canaria*	Passeriformes	wgs (megablast)	CAVT010036532.1	1e−26	100	122
*Sturnus vulgaris*	Passeriformes	wgs (megablast)	LNCF01015430.1	6e−37	100	156
*Taeniopygia guttata*	Passeriformes	wgs (megablast)	VOHI02000025.1 VOHH01000198.1 STFU02000147.1 JAAIVQ010003521.1 JAAIVO010000272.1 JAAIVL010000028.1	1e−24	100	119
*Zosterops lateralis melanops*	Passeriformes	wgs (megablast)	LAII01000220.1	3e−35	100	150
*Egretta garzetta*	Pelecaniformes	wgs (discontiguous megablast)	JJRC01021325.1	1e−13	72	78.8
*Nipponia nippon*	Pelecaniformes	wgs (megablast)	JMFH01044201.1	1e−28	100	128
*Pelecanus crispus*	Pelecaniformes	wgs (megablast)	JJRG01025832.1	2e−21	74	104
*Fulmarus glacialis*	Procellariiformes	wgs (blastn)	JJRN01102231.1	6e−24	100	113
*Aptenodytes forsteri*	Sphenisciformes	wgs (discontiguous megablast)	JMFQ01050174.1	5e−25	100	117
*Tyto alba*	Strigiformes	wgs (blastn)	JJRD01058060.1 CABFVR010017320.1	1e−16	96	90.6
*Phalacrocorax carbo*	Suliformes	wgs (blastn)	JMFI01031110.1	7e−17	94	90.6

Date of search: July 9, 2020.

*wgs* whole-genome shotgun contigs.

### Tissue distribution and diurnal expression pattern

**Figure 2:**
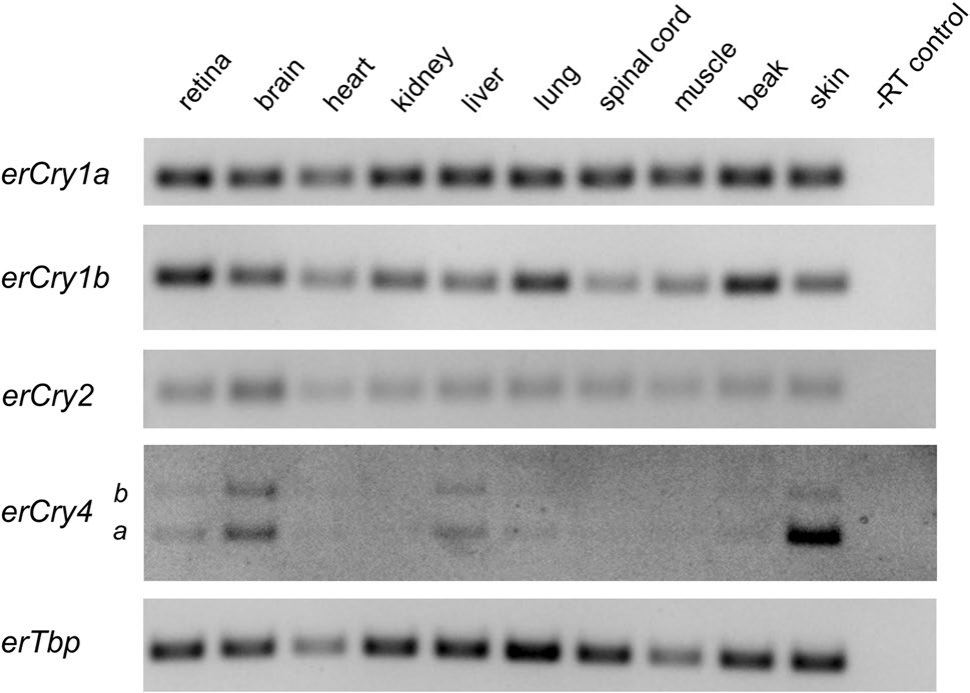
mRNA expression of the five different cryptochromes in various tissues from one European robin. The abundance of cryptochrome mRNA was measured by RT-PCR with cDNA from ten different tissues taken from one bird individual, and the −RT control as a negative control (see “Materials and methods”). The TATA-box binding protein (*erTbp*) served as a reference gene. Full-length gels are provided in the supplement (Fig. S1).

Using RT-PCR, we monitored the mRNA expression pattern of all five cryptochromes known so far in ten European robin tissues (retina, brain, heart, kidney, liver, lung, spinal cord, muscle, beak, and skin), with the TATA-box binding protein (*Tbp*) as a reference. All cryptochromes were found to be expressed in all European robin tissues examined, even if the expression of the two Cry4 transcripts was extremely low in some of the tissues (heart, kidney, spinal cord and muscle) (Fig. 2 and Fig. S1).

**Figure 3:**
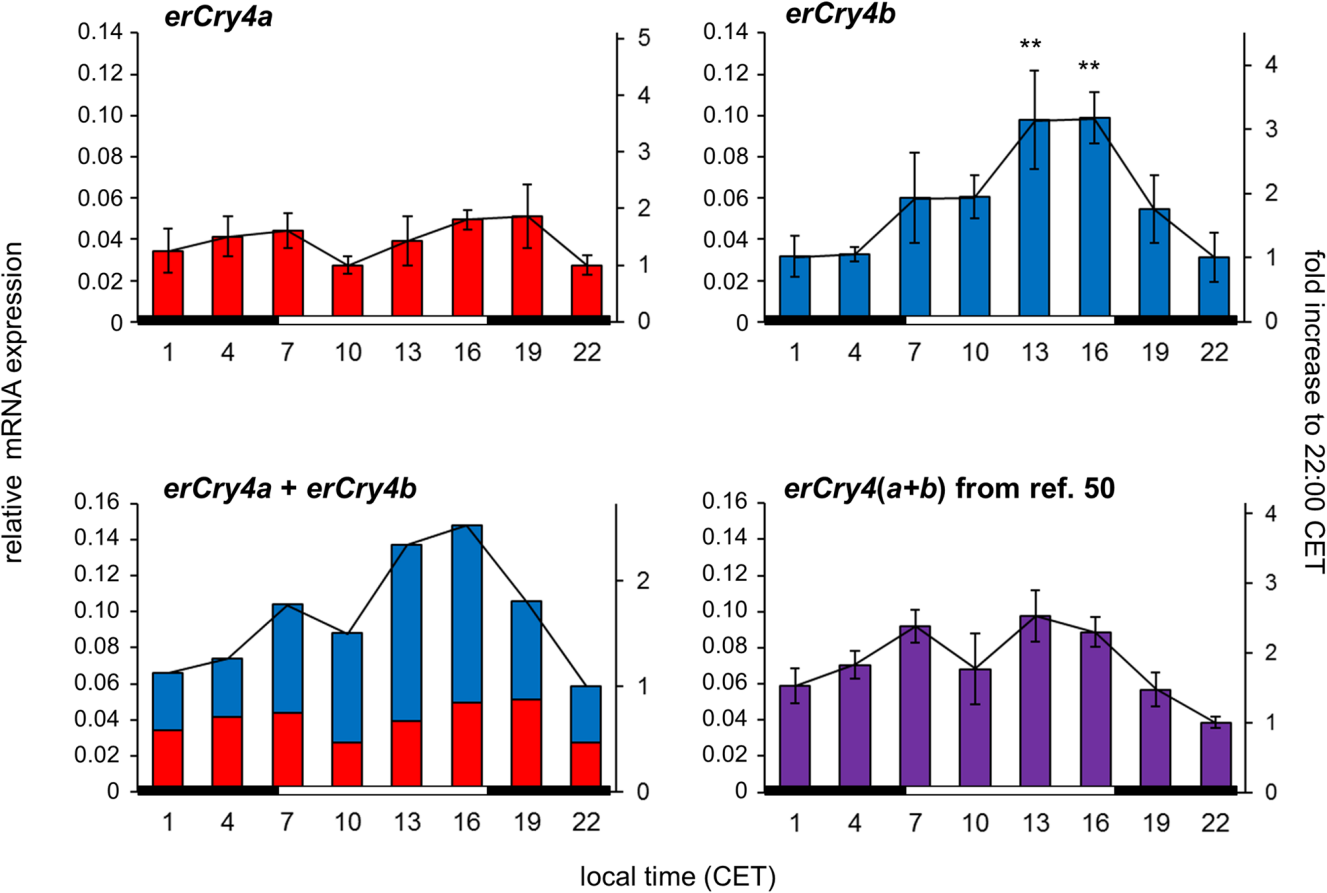
The daily mRNA expression pattern of the erCry4 isoforms *erCry4a* and *erCry4b*. 24-h profiles were measured in retinal tissue collected from three different individuals at eight time points (local time, CET) during the autumn migratory season. Each bar indicates the relative mRNA expression level normalised to the mRNA levels of *erPrkca*, *erGluR2*, and *erTbp*, which were used as reference genes (n = 3, mean values ± SEM; left y axis), and the mRNA amount in relation to the lowest expression value (right y axis). Significant differences at each time point were analysed by one-way factorial ANOVA, p < 0.05; post hoc Tukey HSD test: **p < 0.01. Only significant differences relative to the lowest level of mRNA expression of the respective gene are shown. Note the different y axes for each graph. Data for *erCry4(a* + *b)* (bottom right) is modified from ref.^50^. The light conditions (light and dark period) are illustrated by the black and white bars below the graphs.

We recently analysed the expression pattern over the course of 24 h in four different European robin retinal cryptochrome transcripts, *erCry1a*, *erCry1b*, *erCry2* and *erCry4*, by quantitative RT-PCR^50^. Here, the mRNA of retinal *erCry4* (which included both isoforms *erCry4a* and *erCry4b*) did not show a pronounced 24-h rhythmicity (Fig. 3, bottom right). Separated examination of the erCry4 isoforms in this study revealed that the transcripts of *erCry4a* and *erCry4b* differ in their expression pattern: While *erCry4a* mRNA did not display a clear and significant 24-h rhythmicity in the retina (Fig. 3, top left; one-way ANOVA: p = 0.152, Cosinor analysis: p = 0.274), we observed a pronounced 24-h rhythm of *erCry4b* mRNA expression (Fig. 3, top right; one-way ANOVA: p < 0.001), with a peak in the afternoon (13:00 and 16:00 CET) and the lowest values at night (22:00, 1:00 and 4:00 CET). The increase in relative expression is about threefold (Tukey-HSD: p < 0.01) and statistically significant rhythmicity of *erCry4b* was confirmed by Cosinor analysis (p < 0.001). As expected, adding the values obtained for *erCry4a* and *erCry4b*, respectively, results in a graph (Fig. 3, bottom left) that is very similar to the pooled *Cry4* mRNA expression pattern from our recent publication^50^ (Fig. 3, bottom right).

## Discussion

Avian Cry4 has been suggested to be the primary magnetoreceptor in the retina of night-migratory birds^31,50^. Here, we report that the *Cry4* gene of the night-migratory European robin is expressed in at least two isoforms, which we then analysed with respect to their distribution in tissues and their 24-h rhythmicity in the retina. The longer isoform, erCry4b, might represent the full-length protein, while the shorter (formerly called erCry4) isoform erCry4a is generated by exon-skipping of exon 3 in the course of an alternative RNA splicing process. The *Cry4b*-specific exon seems to be conserved among birds since we also detected it in the retinae of the night-migratory Eurasian blackcap, the non-migratory songbird zebra finch and possibly the domestic chicken. Moreover, it is present in the genome of the majority of the bird species contained in BLAST databases. We found a diverging 24-h expression pattern of retinal *erCry4a* and *erCry4b* mRNA: While *erCry4a* seems to be stably expressed and shows no clear rhythmicity in the retina, *erCry4b* displays a significant 24-h oscillation with its peak in the afternoon (16:00), which corresponds to the expression maximum of both *erCry1a* and *erCry1b* mRNA^50^. The differences in the 24-h expression pattern of the two *erCry4* isoforms likely explain why we only detected a subtle rhythmicity in our recent study that did not distinguish between *erCry4a* and *erCry4b* mRNA^50^. However, we cannot make any predictions about how and when the proteins are functionally active since we only look at the mRNA production level with the qRT-PCR method applied in this study.

When comparing the mRNA levels of the two *Cry4* isoforms in Figs. 1B, 2 and 3, there is an inconsistency of the relative abundancy of *Cry4a* and *Cry4b*: In Figs. 1B and 2, the mRNA levels of *Cry4b* seem to be higher than those of *Cry4a* in Fig. 3. This inconsistency is most likely due to the different primer pairs used for RT-PCR (the same oligonucleotides detected *Cry4a* and *Cry4b*; Figs. 1B and 2) and qRT-PCR (specific primers were used to detect the isoforms separately; Fig. 3), respectively (see Tables 2 and 3). Moreover, although *Cry4b* is mainly expressed during the day, the absolute abundance of the *Cry4a* mRNA at this time point might still be higher.

**Table 2:** Primer sequences and predicted size of the amplified product for the different genes examined for the tissue-wide distribution study.

Gene name	Primer sequence (Fw) 5′–3′	Primer sequence (Rev) 5′–3′	Product length (bp)
*erCry1a*	attgcaagcaggaagaacgg	aatttgtgctctgtcgctgg	113
*erCry1b*	gtctgcaacatgaactgacct	cctcccactcaaaacacgga	119
*erCry2*	ccctgccctgaagaactgta	tcacaaactcttcccaggga	78
*erCry4(a* + *b)*	ccattaacttacaagaggttccttc	tcagtctcccctcctctcca	200 (*erCry4a*) 287 (*erCry4b*)
*TATA box binding protein (erTbp)*	ggcagcaaggaagtatgcaa	ctgaactgctggtgtgtgag	147

**Table 3:** Primer sequences, predicted size of the amplified product and primer pair efficiency for the different genes examined in qRT-PCR.

Gene name	Primer sequence (Fw) 5′–3′	Primer sequence (Rev) 5′–3′	Product length (bp)	Primer pair efficiency
*erCry4a*	tctgtctctgcttggtgacc	ggggcactacatctctggaa	75	2.00
*erCry4b*	agggattgagctcgtgtctc	acatctcctctcctcatgcag	89	2.00
*Protein kinase C alpha (Prkca)*	tgaaggctgaagtcactggt	aagggtctgaaagtccatttgg	93	1.94
*TATA box binding protein (Tbp)*	ggcagcaaggaagtatgcaa	ctgaactgctggtgtgtgag	147	1.99
*Glutamate Receptor 2 (GluR2)*	gtgattccaaggagaagaccag	ccccgacgagaatgtagaaga	72	1.94

Cry4 has been proposed to be the putative primary magnetoreceptor underlying the avian magnetic compass^50,66^. In contrast to Cry1 and Cry2, which are most probably unable to bind the cofactor FAD required for the suggested radical-pair mechanism of magnetoreception^62,69^, Cry4(a) from several species has been shown to bind FAD well^62–64^. Our present finding of a second Cry4 isoform (here named Cry4b), however, raises the question whether both or only one of them might be involved in magnetoreception. The fact that they display a different 24-h mRNA expression pattern in the retina suggests that erCry4a and erCry4b have different functions within the retina. The lack of 24-h rhythmicity of the *erCry4a* mRNA is consistent with what would be predicted for a stably expressed retinal magnetoreceptor. On the other hand, erCry4b, displaying a clear diurnal mRNA oscillation, might have a role diverging from Cry4a function: It could either be a component of the circadian clock, share a putative magnetoreceptor function with erCry4a, or play a completely different, so-far unknown role in the retina. Future studies involving functional expression of Cry4a and Cry4b proteins followed by comparative analysis of their photoreactions could potentially answer this question.

We also examined the tissue-wide mRNA distribution of all known cryptochromes in the European robin and detected a widespread expression of all cryptochromes in all tissues examined. This result is consistent with very recent findings of *clCry1*, *clCry2* and *clCry4* in eleven tissues of the pigeon^65^. The ubiquitous tissue expression of *erCry1a*, *erCry1b* and *erCry2* is not in conflict with their probable role in the circadian clock, since both mammalian Cry1 and Cry2^70^ as well as other circadian clock proteins have also been observed to be widely expressed throughout mammalian tissues^71–73^. A universal tissue distribution of an assumed primary magnetoreceptor, Cry4, on the other side, which is thought to play its key role in the avian retina, seems not very plausible at first sight. However, many proteins perform multiple functions^74^. This phenomenon, termed ‘protein moonlighting’ or ‘gene sharing’, means that one gene can produce proteins with several different molecular functions^74–76^. The moonlighting effect may manifest itself through expression in different cell types or different locations within a cell^75^. Not only might one and the same Cry4 isoform play different roles in different tissues, but both Cry4 isoforms could also have different functions (both within the retina and throughout the body). To find out more about possibly diverging roles of the two Cry4 isoforms in the avian retina, it will be necessary to produce antibodies specifically recognising either Cry4a or Cry4b protein.

In conclusion, we found an additional isoform (Cry4b) of the putative magnetoreceptor Cry4 in the retina of several bird species. The mRNA of this novel isoform is primarily expressed during the day whereas the mRNA of the formerly known Cry4(a) is expressed at similar level throughout a 24-h period. This could indicate that the two isoforms play different roles within both the retina and/or the whole avian organism.

## Materials and methods

### Birds

Twenty-six European robins (*Erithacus rubecula*) and two Eurasian blackcaps (*Sylvia atricapilla*) were wild-caught in the vicinity of the campus of the University of Oldenburg using mist nets. Two zebra finches (*Taeniopygia guttata*) were captive-bred. Two chickens (*Gallus gallus domesticus*) were bred from specific-pathogen-free eggs (VALO Biomedia, Osterholz-Scharmbeck, Germany) and raised in the animal care facility of the University of Oldenburg. Of the 26 European robins used in the qRT-PCR study, 24 were the same that were used in ref.^50^ (Table S1, bird number 1–24 in ref.^50^). All animal procedures were performed in accordance with local and national guidelines for the use of animals in research and were approved by the Animal Care and Use Committees of the Niedersächsisches Landesamt für Verbraucherschutz und Lebensmittelsicherheit (LAVES, Oldenburg, Germany), Az: 3314-42502-04-10/0121.

### Tissue collection, RNA isolation and cDNA production

The tissues were collected from birds sacrificed by decapitation. One retina from each bird, free of vitreous, and the other tissues used in this study were put into ice-cold TRIzol Reagent (Life Technologies, Carlsbad, CA, USA), shock-frozen in liquid nitrogen and stored at − 80 °C until RNA extraction. The total RNA was isolated according to manufacturer’s instructions. RNA concentration was measured using the Infinite 200 PRO instrument (Tecan, Männedorf, Switzerland). RNA quality was determined with the Agilent RNA 6000 Nano Kit using a 2100 Bioanalyzer Instrument (Agilent Technologies, Santa Clara, CA, USA). For each sample, 1 μg total RNA was reverse-transcribed using the QuantiTect Reverse Transcription Kit (Qiagen, Hilden, Germany), which included a genomic DNA removal step, according to the manufacturer’s instructions. As a negative control, samples from each batch of reverse transcription were pooled and incubated with primer mix lacking reverse transcriptase (− RT control). The obtained cDNA was diluted 1/20 in 0.1 × TE buffer and stored at − 20 °C until use.

### Amplification and cloning of the erCry4b cDNA sequence

Specific primers with restriction sites were designed to amplify the coding region of the European robin erCry4 mRNA sequence (gene bank accession number KX890129^50^). The primers erCry4-Xho1-pT-AE-F, 5-ggtattctcgagatgctgcatcgcaccat-3′ and erCry4-KpnI-pT-AE-R, 5′-tattggtaccgtgtattctgttgttcggg-3′ were used for polymerase chain reaction (PCR) amplification with the Phusion High-Fidelity DNA Polymerase (Thermo Fisher Scientific, Waltham, MA, USA; Cat. No. F-530XL) following the manufacturer’s instructions. The cycle conditions were: one cycle denaturation at 98 °C (30 s), 30 cycles at 98 °C (10 s), 64 °C (30 s), 72 °C (60 s), with a final extension of 10 min at 72 °C. The PCR product was run on a 2% agarose gel for 120 to 150 min at 90 V. The two *erCry4* isoforms *erCry4a* and *erCry4b* had separated on the gel due to their size difference. The PCR products were cut out from the gel, purified using the QIAquick Gel Extraction Kit (Qiagen) and subjected to sequencing (LGC, Berlin, Germany). Sequence analysis was carried out using Clustal Omega from the European Bioinformatics Institute (https://www.ebi.ac.uk/Tools/msa/clustalo/). After detection of a longer isoform (*erCry4b*), the respective PCR product was digested with the XhoI/KpnI Fast Digest restriction enzymes (Thermo Fisher Scientific) and cloned into the expression vector pTurboGFP-N (Evrogen, Moscow, Russia) according to the protocol for the Rapid DNA Dephos & Ligation Kit (Roche Diagnostics, Rotkreuz, Switzerland). To amplify *erCry4b* from the retinae of European robins, Eurasian blackcaps, zebra finches and domestic chickens (Fig. 1B), the primers Cry4-F493 (5′-gaggtgcctgtccgaaac-3′) and Cry4-R835 (5′-tcaggcctgtggtgcttg-3′) were used in a PCR reaction with the GoTaq Long PCR Master Mix (Promega), which included pre-incubation at 95 °C for 5 min, followed by 36 cycles of denaturation at 94 °C for 15 s, annealing at 56 °C for 30 s and extension at 72 °C for 30 s, with a final incubation at 72 °C for 10 min. The expected product sizes were 254 bp for Cry4a and 343 bp for Cry4b. Ten µl of a 25 µl PCR amplification assay were subsequently analysed on a 1.7% agarose gel with the BioRad QuickLadder 100 bp as ladder.

### RT-PCR tissue distribution

We performed RT-PCR to characterize the gene expression of all known cryptochromes in ten different tissues. The method of RNA extraction and cDNA synthesis was the same as described in the section ‘Tissue collection, RNA isolation and cDNA production’. The RNA from all tissues had been subjected to quality analysis using a Bioanalyzer 2000 (Agilent), and only RNA with a RIN > 7.9 was used for cDNA synthesis. Specific primers (listed in Table 2) were designed using Primer 3 (https://bioinfo.ut.ee/primer3-0.4.0/). PCR was performed using GoTaq Long PCR Master Mix (Promega, Madison, WI, USA), which included pre-incubation at 95 °C for 5 min, followed by 32 cycles of denaturation at 94 °C for 15 s, annealing at 56 °C for 30 s and extension at 72 °C for 30 s, with a final incubation at 72 °C for 10 min. Seven µl of a 25 µl PCR sample were run on a 2% agarose gel for 90 min at 70 V.

### Quantitative reverse transcription (qRT)-PCR

The qRT-PCR was performed as described before^50^. Primer sequences are listed in Table 3. Shortly, 1/400 of the total cDNA yield (2.5 ng cDNA) was used for each qPCR reaction, which contained 1 µl of a 2 µM primer solution, 5 μl FastStart Essential DNA Green Master (Roche) and 1 µl cDNA (diluted product; see above) in a 10 μl reaction volume. All samples were run in triplicate. The reaction was run at default settings (pre-incubation 95 °C, 600 s; 3 step amplification 95 °C, 10 s; 55 °C, 10 s; 72 °C, 10 s; 45 cycles) on the LightCycler 96 Instrument (Roche). Reference genes (listed in Table 3) had been selected for between-sample normalisation^50^.

### Statistical analysis

The statistical analysis of the 24-h expression data was performed as described before^50^. All results are presented as means ± SEM. To test for a statistically significant difference among the different groups, the p value of ANOVA was calculated based on the normalized data (SPSS package 23, SPSS Inc., IL, USA), with Tukey HSD as post hoc test. To evaluate the rhythmicity in gene expression, the Cosinor method was performed with software available at https://www.circadian.org/softwar.html. The expression was considered to display a 24-h rhythmicity if it had both p < 0.05 by ANOVA and SE(A)/A < 0.3 by Cosinor analysis.

## Supplementary information

Supplementary file1

## References

[1] Mouritsen H (2018). Long-distance navigation and magnetoreception in migratory animals. Nature.

[2] Frost BJ, Mouritsen H (2006). The neural mechanisms of long distance animal navigation. Curr. Opin. Neurobiol..

[3] Middendorff, A. T. von. Die Isepiptesen Russlands. Grundlagen zur Erforschung der Zugzeiten und Zugrichtungen der Vögel Russlands. *Mem. Acad. Sci. St. Petersbourg VI, Ser. Tome.* **8**, 1–143 (1855).

[4] Merkel FW, Wiltschko W (1965). Magnetismus und Richtungsfinden zugunruhiger Rotkehlchen (*Erithacus rubecula*). Vogelwarte.

[5] Wiltschko W (1968). Über den Einfluß statischer Magnetfelder auf die Zugorientierung der Rotkehlchen, Erithacus rubecula. Z. Tierpsychol..

[6] Wiltschko W, Wiltschko R (1972). Magnetic compass of European robins. Science.

[7] Wiltschko W, Wiltschko R (1995). Migratory orientation of European robins is affected by the wavelength of light as well as by a magnetic pulse. J. Comp. Physiol. A.

[8] Zapka M (2009). Visual but not trigeminal mediation of magnetic compass information in a migratory bird. Nature.

[9] Hein CM (2010). Night-migratory garden warblers can orient with their magnetic compass using the left, the right or both eyes. J. R. Soc. Interface.

[10] Hein CM, Engels S, Kishkinev D, Mouritsen H (2011). Robins have a magnetic compass in both eyes. Nature.

[11] Engels S, Hein CM, Lefeldt N, Prior H, Mouritsen H (2012). Night-migratory songbirds possess a magnetic compass in both eyes. PLoS ONE.

[12] Wiltschko W, Munro U, Ford H, Wiltschko R (1993). Red-light disrupts magnetic orientation of migratory birds. Nature.

[13] Muheim R, Bäckman J, Åkesson S (2002). Magnetic compass orientation in European robins is dependent on both wavelenght and intensity of light. J. Exp. Biol..

[14] Wiltschko W, Gesson M, Wiltschko R (2001). Magnetic compass orientation of European robins under 565 nm green light. Naturwissenschaften.

[15] Wiltschko W, Möller A, Gesson M, Noll C, Wiltschko R (2004). Light-dependent magnetoreception in birds: analysis of the behaviour under red light after pre-exposure to red light. J. Exp. Biol..

[16] Heyers D, Manns M, Luksch H, Güntürkün O, Mouritsen H (2007). A visual pathway links brain structures active during magnetic compass orientation in migratory birds. PLoS ONE.

[17] Zapka M, Heyers D, Liedvogel M, Jarvis ED, Mouritsen H (2010). Night-time neuronal activation of Cluster N in a day- and night-migrating songbird. Eur. J. Neurosci..

[18] Mouritsen H, Feenders G, Liedvogel M, Wada K, Jarvis ED (2005). Night-vision brain area in migratory songbirds. Proc. Natl. Acad. Sci. USA.

[19] Mouritsen H, Heyers D, Güntürkün O (2016). The neural basis of long-distance navigation in birds. Annu. Rev. Physiol..

[20] Wiltschko W (2007). The magnetic compass of domestic chickens, Gallus gallus. J. Exp. Biol..

[21] Denzau S, Nießner C, Rogers LJ, Wiltschko W (2013). Ontogenetic development of magnetic compass orientation in domestic chickens (Gallus gallus). J. Exp. Biol..

[22] Keary N (2009). Oscillating magnetic field disrupts magnetic orientation in Zebra finches, *Taeniopygia guttata*. Front. Zool..

[23] Pinzon-Rodriguez A, Muheim R (2017). Zebra finches have a light-dependent magnetic compass similar to migratory birds. J. Exp. Biol..

[24] Liedvogel M, Mouritsen H (2009). Cryptochromes—a potential magnetoreceptor: what do we know and what do we want to know?. J. R. Soc. Interface.

[25] Mouritsen H, Scanes C (2015). Magnetoreception in birds and its use for long distance migration. Sturkie’s Avian Physiology.

[26] Schulten K, Swenberg CE, Weller A (1978). A biomagnetic sensory mechanism based on magnetic field modulated coherent electron spin motion. Z. Phys. Chem..

[27] Ritz T, Adem S, Schulten K (2000). A model for photoreceptor-based magnetoreception in birds. Biophys. J..

[28] Maeda K (2008). Chemical compass model of avian magnetoreception. Nature.

[29] Engels S (2014). Anthropogenic electromagnetic noise disrupts magnetic compass orientation in a migratory bird. Nature.

[30] Schwarze S (2016). Weak broadband electromagnetic fields are more disruptive to magnetic compass orientation in a night-migratory songbird (*Erithacus rubecula*) than strong narrow-band fields. Front. Behav. Neurosci..

[31] Hore PJ, Mouritsen H (2016). The radical-pair mechanism of magnetoreception. Annu. Rev. Biophys..

[32] Hiscock HG (2016). The quantum needle of the avian magnetic compass. Proc. Natl Acad. Sci. USA.

[33] Ritz T, Thalau P, Phillips JB, Wiltschko R, Wiltschko W (2004). Resonance effects indicate a radical pair mechanism for avian magnetic compass. Nature.

[34] Ritz T (2009). Magnetic compass of birds is based on a molecule with optimal directional sensitivity. Biophys. J..

[35] Kavokin K (2014). Magnetic orientation of garden warblers (Sylvia borin) under 1.4 MHz radiofrequency magnetic field. J. R. Soc. Interface.

[36] Ahmad M, Cashmore AR (1993). HY4 gene of A. thaliana encodes a protein with characteristics of a blue-light photoreceptor. Nature.

[37] Giovani B, Byrdin M, Ahmad M, Brettel K (2003). Light-induced electron transfer in a cryptochrome blue-light photoreceptor. Nat. Struct. Biol..

[38] Zeugner A (2005). Light-induced electron transfer in Arabidopsis cryptochrome-1 correlates with in vivo function. J. Biol. Chem..

[39] Biskup T (2009). Direct observation of a photoinduced radical pair in a cryptochrome blue-light photoreceptor. Angew. Chem. Int. Ed. Engl..

[40] Dodson CA, Hore PJ, Wallace MI (2013). A radical sense of direction: signalling and mechanism in cryptochrome magnetoreception. Trends Biochem. Sci..

[41] Sancar A (2003). Structure and function of DNA photolyase and cryptochrome blue-light photoreceptors. Chem. Rev..

[42] Lin CT, Todo T (2005). The cryptochromes. Genome Biol..

[43] Chaves I (2011). The cryptochromes: blue light photoreceptors in plants and animals. Annu. Rev. Plant Biol..

[44] Michael AK, Fribourgh JL, Van Gelder RN, Partch CL (2017). Animal cryptochromes: divergent roles in light perception, circadian timekeeping and beyond. Photochem. Photobiol..

[45] Nießner C (2011). Avian ultraviolet/violet cones identified as probable magnetoreceptors. PLoS ONE.

[46] Bolte P (2016). Localisation of the putative magnetoreceptive protein cryptochrome 1b in the retinae of migratory birds and homing pigeons. PLoS ONE.

[47] Nießner C (2016). Seasonally changing cryptochrome 1b expression in the retinal ganglion cells of a migrating passerine bird. PLoS ONE.

[48] Möller A, Sagasser S, Wiltschko W, Schierwater B (2004). Retinal cryptochrome in a migratory passerine bird: a possible transducer for the avian magnetic compass. Naturwissenschaften.

[49] Mouritsen H (2004). Cryptochromes and neuronal-activity markers colocalize in the retina of migratory birds during magnetic orientation. Proc. Natl. Acad. Sci. USA.

[50] Günther A (2018). Double-cone localization and seasonal expression pattern suggest a role in magnetoreception for European robin cryptochrome 4. Curr. Biol..

[51] Kobayashi Y (2000). Molecular analysis of zebrafish photolyase/cryptochrome family: two types of cryptochromes present in zebrafish. Genes Cells.

[52] Kubo Y, Akiyama M, Fukada Y, Okano T (2006). Molecular cloning, mRNA expression, and immunocytochemical localization of a putative blue-light photoreceptor CRY4 in the chicken pineal gland. J. Neurochem..

[53] Watari R (2012). Light-dependent structural change of chicken retinal Cryptochrome4. J. Biol. Chem..

[54] Takeuchi T, Kubo Y, Okano K, Okano T (2014). Identification and characterization of cryptochrome4 in the ovary of western clawed frog *Xenopus tropicalis*. Zoolog. Sci..

[55] Liu C (2015). Molecular evolution and functional divergence of zebrafish (*Danio rerio*) cryptochrome genes. Sci. Rep..

[56] Wiltschko R, Wiltschko W (1995). Magnetic Orientation in Animals (Zoophysiology Volume 33).

[57] Phillips JB (1996). Magnetic navigation. J. Theor. Biol..

[58] Putman NF, Endres CS, Lohmann CM, Lohmann KJ (2011). Longitude perception and bicoordinate magnetic maps in sea turtles. Curr. Biol..

[59] Putman NF (2013). Evidence for geomagnetic imprinting as a homing mechanism in Pacific salmon. Curr. Biol..

[60] Mouritsen H, Galizia CG, Lledo PM (2013). The magnetic senses. Neurosciences— From Molecule to Behavior: A University Textbook.

[61] Bottesch M (2016). A magnetic compass that might help coral reef fish larvae return to their natal reef. Curr. Biol..

[62] Öztürk N (2009). Comparative photochemistry of animal type 1 and type 4 cryptochromes. Biochemistry.

[63] Mitsui H (2015). Overexpression in yeast, photocycle, and in vitro structural change of an avian putative magnetoreceptor cryptochrome4. Biochemistry.

[64] Qin S (2016). A magnetic protein biocompass. Nat. Mater..

[65] Wang X (2018). Comparative properties and functions of type 2 and type 4 pigeon cryptochromes. Cell. Mol. Life Sci..

[66] Zoltowski BD (2019). Chemical and structural analysis of a photoactive vertebrate cryptochrome from pigeon. Proc. Natl. Acad. Sci. USA.

[67] Wu H, Scholten A, Einwich A, Mouritsen H, Koch KW (2020). Protein-protein interaction of the putative magnetoreceptor cryptochrome 4 expressed in the avian retina. Sci. Rep..

[68] Wang ET (2008). Alternative isoform regulation in human tissue transcriptomes. Nature.

[69] Kutta RJ, Archipowa N, Johannissen LO, Jones AR, Scrutton NS (2017). Vertebrate cryptochromes are vestigial flavoproteins. Sci. Rep..

[70] Miyamoto Y, Sancar A (1998). Vitamin B2-based blue-light photoreceptors in the retinohypothalamic tract as the photoactive pigments for setting the circadian clock in mammals. Proc. Natl. Acad. Sci. USA.

[71] Plautz JD, Kaneko M, Hall JC, Kay SA (1997). Independent photoreceptive circadian clocks throughout Drosophila. Science.

[72] Hege DM, Stranewsky R, Hall JC, Giebultowicz JM (1997). Rhythmic expression of a PER-reporter in the Malpighian tubules of decapitated Drosophila: evidence for a brain-independent circadian clock. J. Biol. Rhythms.

[73] Giebultowicz JM, Hege DM (1997). Circadian clock in Malpighian tubules. Nature.

[74] Jeffery CJ (2020). Enzymes, pseudoenzymes, and moonlighting proteins: diversity of function in protein superfamilies. FEBS J..

[75] Jeffery CJ (1999). Moonlighting proteins. Trends Biochem. Sci..

[76] Piatigorsky J (2007). Gene Sharing and Evolution. The Diversity of Protein Functions.

