# Supplementary material for: A novel isoform of cryptochrome 4 (Cry4b) is expressed in the retina of a night-migratory songbird
Source: Sci Rep. 2020 Sep 25;10:15794. doi: 10.1038/s41598-020-72579-2 (PMC7519125; doi:10.1038/s41598-020-72579-2)
Supplement: Supplementary file 1 — Supplementary file1 [file 41598_2020_72579_MOESM1_ESM.docx]

***Supplementary information***

**A novel isoform of cryptochrome 4 (Cry4b) is expressed in the retina of a night-migratory songbird**

Angelika Einwich^1,2^, Karin Dedek^1,2^, Pranav Kumar Seth^1,2^, Sascha Laubinger^3^, Henrik Mouritsen^1,2,*^

^1^Institute for Biology and Environmental Sciences, Neurosensorics/Animal Navigation, Carl-von-Ossietzky-University Oldenburg, Oldenburg, Germany

^2^Research Centre for Neurosensory Sciences, Carl-von-Ossietzky-University Oldenburg, Oldenburg, Germany

^3^Institute for Biology and Environmental Sciences, Evolutionäre Genetik der Pflanzen, Carl-von-Ossietzky-University Oldenburg, Oldenburg, Germany


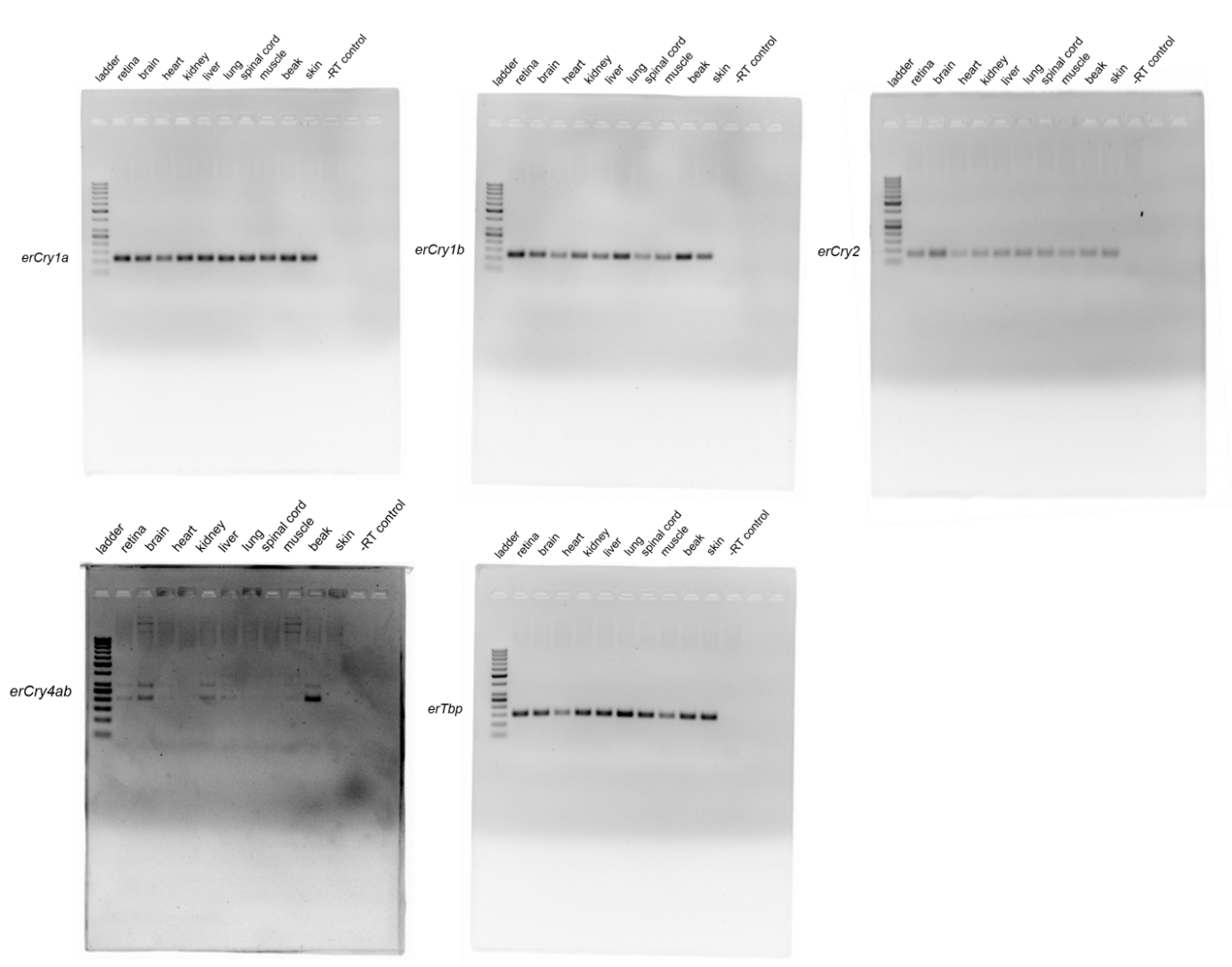


**Figure S1. Full-lengths PCR gels (related to Fig. 2).**
